# Diagnostic anténatal de la dysplasie rénale multikystique: à propos de 18 cas

**DOI:** 10.11604/pamj.2019.33.279.18485

**Published:** 2019-07-31

**Authors:** Hekmat Chaara, Hind Adadi, Imane Attar, Sofia Jayi, Fatima-Zahra Fdili Alaoui, Moulay Abdelilah Melhouf

**Affiliations:** 1 Service Gynécologie Obstétrique II, CHU Hassan II, Fès, Maroc

**Keywords:** Diagnostic anténatal, dysplasie rénale multikystique, échographie, pronostic, Prenatal diagnosis, multicystic dysplastic kidney, ultrasound, prognosis

## Abstract

La dysplasie rénale multikystique (DRMK) correspond à l'expression clinique la plus fréquente des “Congenital Abnormalities of Kidney and Urinary Tract (CAKUT)”. Son étiopathogénie reste discutée et l'hypothèse obstructive est généralement admise. L'échographie obstétricale représente le moyen de référence pour son diagnostic anténatal et qui nécessitera la recherche d'autres malformations associées. Une prise en charge anténatale repose principalement sur la surveillance échographique de l'évolution de la grossesse et de la pathologie, de l'apparition d'autres anomalies et de la quantité du liquide amniotique. L'objectif est de rapporter l'expérience du Service de Gynécologie-Obstétrique 2 du CHU Hassan II de Fès afin de: préciser l'épidémiologie de la DRMK; rapporter la pertinence de l'échographie obstétricale dans le diagnostic positif et étiologique; décrire les aspects échographiques; établir un suivi des grossesses et évaluer l'évolution de la pathologie; écrire les modalités de surveillance et de prise en charge anténatales; rapporter l'évolution en postnatal et établir un pronostic.

## Abstract (en)

Multicystic dysplastic kidney (MCDK) is the most common Congenital Abnormalities of Kidney and Urinary Tract (CAKUT) in clinical practice. Its etiology and pathogenesis are still controversial and obstruction is a generally accepted causative assumption. Obstetric ultrasound is the gold standard for prenatal diagnosis and for the detection of other associated malformations. Prenatal management is based on ultrasound monitoring of pregnancy, of the disease, of the occurrence of other anomalies and of the amount of amniotic fluid. This study aims to report our experience in the Department of Gynecology-Obstetrics 2 at the University Hospital Hassan II-Fez in order to clarify the epidemiology of MCDK, highlight the role of obstetric ultrasound in positive and etiological diagnosis while describing the various sonographic appearances. It is essential to schedule for screening tests during pregnancy in order to assess the evolution of the disease diagnosed in the prenatal period, to implement a strategy in postpartum care and to establish a prognosis.

## Introduction

La dysplasie rénale multikystique (DRMK) est une des plus fréquentes malformations de l'appareil urinaire, regroupées sous le terme de “Congenital Abnormalities of Kidney and Urinary Tract (CAKUT)”, ou anomalies congénitales des reins et des voies urinaires. Sa pathogénie est incomplètement élucidée. Dans la vision classique, la DRMK résulterait d'un défaut d'induction du blastème métanéphrique par le bourgeon urétéral. Une autre hypothèse suggère que la DRMK serait secondaire à une anomalie majeure de l'écoulement des urines fœtales survenant précocement dans le développement rénal. C'est une pathologie sans transmission génétique, sporadique, définie par l'association de kystes parenchymateux à une dysplasie rénale vraie homolatérale (existence de tubes primitifs et de métaplasie cartilagineuse à l'examen histologique). L'incidence de la dysplasie rénale multikystique est estimée à une sur 4300 naissances. Elle est décrite comme le plus souvent unilatérale (75%) avec une localisation au niveau du rein gauche dans 53% des cas. Alors que la forme bilatérale est généralement léthale. Elle touche le sexe masculin dans 60% des cas. Le diagnostic anténatal de la DRMK repose essentiellement sur l'échographie obstétricale. L'intérêt de ce dépistage est évident, il permet de: détecter une anomalie qui pourrait passer inaperçue à la naissance et ne se dévoiler que tardivement lorsque l'atteinte rénale est devenue irréversible; rechercher des anomalies rénales ou extra-rénales associées.

## Méthodes

Notre travail est une étude rétrospective étalée sur une période de 7 ans s'étendant de janvier 2011 à décembre 2017 et portant sur une série de 18 cas de parturientes suspectées d'être porteuse d'un fœtus atteint de dysplasie rénale multikystique au sein de notre service.

## Résultats

**Epidémiologie**: l'incidence annuelle a augmenté depuis l'instauration de l'unité de diagnostic anténatal avec une moyenne de 2,6 nouveaux cas par an. Les 71.43% des fœtus étaient de sexe masculine pour seulement 28,57% de sexe féminin. Dans notre série, seulement 16% de nos patientes avaient des antécédents familiaux de pathologie de l'arbre urinaire à type de: insuffisance rénale terminale, duplicité rénale.

### Détails du diagnostic anténatal

**Biométrie**: dans notre série, huit fœtus porteurs de DRMK présentaient un retard de croissance intra-utérin. Parmi eux, quatre étaient atteint d'une DRMK unilatérale et les quatre autres de DRMK bilatérale. L'atteinte unilatérale du rein ne peut vraisemblablement pas expliquer à elle seule le retard de croissance intra-utérin (RCIU). Pour cela, il faut rechercher un contexte auquel le RCIU pourrait être rattaché. 3/4 des DRMK unilatérales avec RCIU de notre série avaient des malformations associées (Tableau 1).

**Tableau 1:** Répartition des cas de DRMK unilatérales selon le contexte du RCIU

DRMK unilatérale	Nombre de cas
**Contexte du RCIU**	
Malformation cérébrale et cardiaque	1
Agénésie du rein controlatéral	1
Trisomie 21	1
Imprécis	1

**Figure 1:**
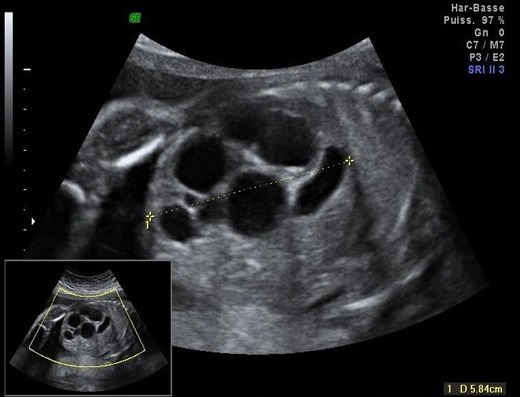
Rein multikystique

**Figure 2:**
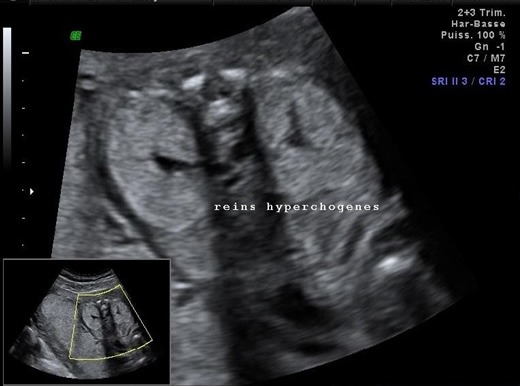
Gros reins hyperéchogènes sans différenciation cortico- médullaire

**Figure 3:**
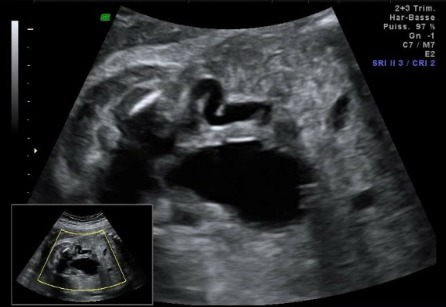
Dilatation urétérale associée à la DRMK

**Morphologie rénale**: à l'échographie, le diagnostic de la DRMK est évident. Dans notre étude, le rein dysplasique et multikystique apparait sous plusieurs aspects différents: 1) rein multikystique avec des kystes de différentes tailles allant de quelques mm à plusieurs cm (Figure 1); 2) les reins atrophiés, de petite taille, sans différentiation cortico-médullaire (Figure 2); 3) des reins hyperéchogènes sièges de kystes corticaux millimétriques. La taille des reins était variable en fonction de la taille et du nombre des kystes qui allaient d'un seul à une dizaine de kystes. L'hypertrophie compensatrice du rein controlatéral a été retrouvé dans 3 cas de DRMK unilatérale.

**Topographie de la dysplasie rénale multikystique**: une prédominance de l'atteinte rénale bilatérale a été objectivée avec un taux de 61% contre 39% pour l'atteinte unilatérale. Le côté gauche est plus fréquemment impliqué quand l'atteinte est unilatérale avec un taux de 27,8. L'atteinte était focale dans un seul cas au niveau du pôle supérieur du rein controlatéral.

**Figure 4:**
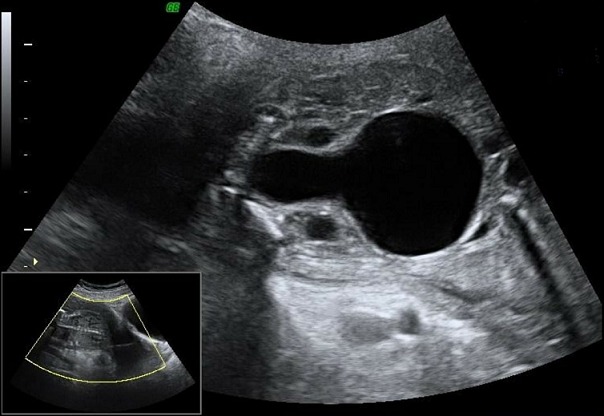
Valve de l’urètre postérieur: mégavessie avec récessus vésical

**Figure 5:**
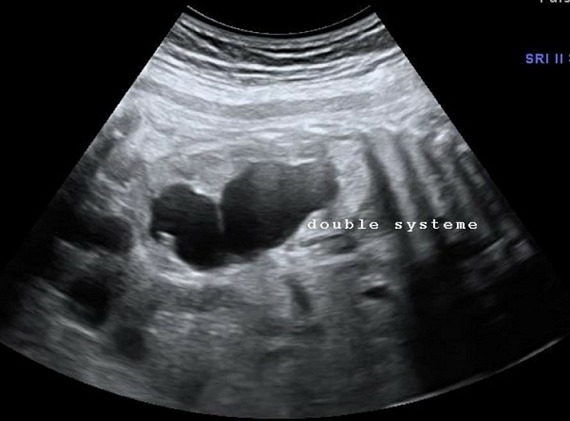
Système double

**Anomalies de l'appareil uro-génital associées**: dans 72,22% des cas, la dysplasie rénale multikystique n'était pas isolée. La dilatation urétéropyélocalcielle était l'anomalie la plus fréquente (Figure 3) avec un pourcentage de presque 40%, suivie de la valve de l'urètre postérieure avec 28% (Figure 4). Dans les DRMK unilatérales, le rein controlatéral était atteint dans 67% des cas, avec 3 cas d'hypertrophie compensatrice, deux cas d'hydronéphrose, deux cas de duplicité (Figure 5) et enfin un cas d'agénésie rénale. On a recensé un cas de DRMK sur des reins pelviens. De plus, la vessie était augmentée de taille dans 3 cas (17%) et dans 11% des cas l'ascite urinaire était présente. On a également diagnostiqué un cas d'hydrocèle (Tableau 2).

**Tableau 2:** Répartition des cas selon les anomalies de l’appareil uro-génital associées à la dysplasie rénale multikystique

Anomalie	Nombre de cas	Pourcentage (%)
Ascite urinaire	2	11,12
Hypertrophie compensatrice du rien controlatéral	3	16,67
Hydronéphrose	2	11,12
Agénésie rénale controlatérale	1	5,56
Ectopie rénale (pelvis)	1	5,56
Dilatation urétéropyélocalicielle	7	38,9
Valve de l’urètre postérieure (VUP)	5	27,77
Système double	2	11,12
Hydrocèle	1	5,56
Mégavessie	3	16,67

**Les malformations extra-rénales associées à la dysplasie rénale multikystique**: (Tableau 3).

**Tableau 3:** Répartition des cas en fonction des anomalies extra-urinaires associées

Appareil fœtal atteint	Nombre de cas	Description	Pourcentage (%)
Système nerveux	3	Agénésie partielle du vermis	16,67
		Encéphalocèle	
		Microcéphalie + anomalie de la FCP	
Face et cou	2	Dysmorphie faciale	11,11
		Lymphangiome kystique	
Système cardio- vasculaire	2	Epanchement péricardique	11,11
		Petit VG	
Système pleuro-pulmonaire	1	Hypoplasie pulmonaire	5,55
Paroi abdominale	1	Flasque et fine	5,55
Membres	2	Fémur court et pieds bots	11,11
		Hexadactylie	

**Figure 6:**
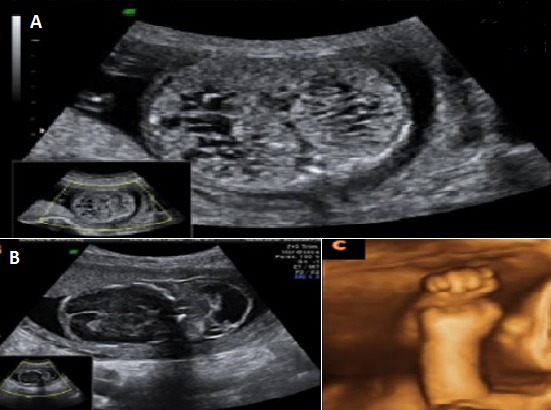
Syndrome de Meckel Grüber: A) gros reins hyperéchogènes avec des kystes médullaires; B): encéphalocèle occipitale; C) reconstruction échographique montrant une hexadactylie

**Figure 7:**
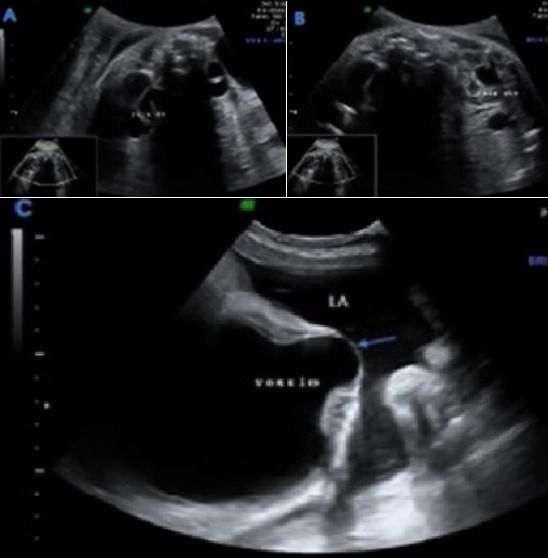
Syndrome de Meckel Grüber: A) gros reins hyperéchogènes avec des kystes médullaires; B) encéphalocèle occipitale; C) reconstruction échographique montrant une hexadactylie

**Regroupement syndromique**: d'après tous les cas étudiés de DRMK et toutes les malformations associées, on a pu regrouper certains sous des formes syndromiques comme tels: 1) syndrome de Down (ou Trisomie 21); 2) la trisomie 21 a été suspectée chez deux cas (11,11% des cas), respectivement à 31 SA et à 22 SA; 3) le premier cas avait retrouvé une dysplasie rénale multikystique droite associée à un Sd polymalformatif avec une agénésie partielle du vermis, un canal atrio-ventriculaire déséquilibré avec un petit ventricule gauche et un hydramnios; 4) le deuxième cas, la dysplasie rénale multikystique gauche était associée à un fémur court et des pieds bots; 5) syndrome de Meckel Grüber (Figure 6). Le syndrome de Meckel Grüber a été évoqué dans deux cas (soit avec un pourcentage de 11,11%) où les reins étaient hyperéchogènes dans les deux cas associés à des malformations cérébrales, faciales et des extrémités. L'âge gestationnel de diagnostic était à 15 SA +5J et à 21 SA +2J. 6) Syndrome de Prune Belly (Figure 7). Ce syndrome a été décrit à 21SA +6J dans un seul cas qui présentait une DRMK bilatérale secondaire probablement à une valve de l'urètre postérieure. La paroi abdominale était flasque paraissant scellée à la paroi utérine.

**Evolution échographique de la DRMK**: on a pu suivre l'évolution de la grossesse chez 15 parturientes alors que les 3 autres ont été référées pour avis spécialisé et donc n'ont bénéficié que d'une seule échographie au sein de notre unité de diagnostic prénatal. L'évolution a été marquée par la stabilisation des atteintes chez 8 cas (44,44 %) et l'aggravation de la dysplasie rénale chez les 7 autres cas (38,9%) soit avec l'augmentation de la taille et du nombre des kystes, l'apparition de kystes au niveau du rein controlatéral ou encore la diminution de la quantité du liquide amniotique. L'évolution défavorable a concerné 4 cas de DRMK unilatérale et 3 cas de DRMK bilatérale (Tableau 4).

**Tableau 4:** Répartition des cas selon le mode d’aggravation de la dysplasie rénale multikystique

Type d’atteinte	DRMK unilatérale	DRMK Bilateral	Totale	
			Nombre de cas	Poucentage (%)
↗ Nombre et taille des Kystes	2	1	3	16,7
Apparition de kystes dans le rein controlatéral	4	-	4	22,22
↙ Quantité de LA	1	2	3	16,7

**Evolution des grossesses**: (Tableau 5).

**Tableau 5:** Évolution de toutes les grossesses de notre étude

	Vivant		Décédé		PDV		IMG		MFIU	
DRMK	Unilatérale	Bilatérale	Unilatérale	Bilatérale	Unilatérale	Bilatérale	Unilatérale	Bilatérale	Unilatérale	Bilatérale
Prématuré	1	0	1	1	0	0	-	-	-	-
A terme	3	2	1	2	1	0	-	-	-	-
**Total: 18**	4	2	2	3	1	**0**	**0**	**3**	**0**	**3**
**(100%)**	**6 (34%)**		**5(2%)**		**1 (6 %)**		**3 (17%)**		**3 (17%)**	

**PDV:** perdu de vue; **IMG:** interruption médicale de grossesse; **MFIU:** mort fœtale in utero

**Evolution des prématurés en post-partum**: (Tableau 6) sur les trois prématurés, deux sont décédés dans la période néonatale: 1) le premier, porteur d'une DRMK associée à une duplicité et un système double, a été hospitalisé en néonatologie pour détresse respiratoire et une insuffisance rénale puis décédé à J23 de vie; 2) le deuxième était atteint d'une DRMK bilatérale et a succombé à H10 de vie. Quant au troisième prématuré, porteur d'une DRMK gauche et chez qui on suspectait une trisomie 21, son évolution était favorable avec une bonne fonction rénale et sans autre anomalie à la naissance sauf des pieds bots pour lesquels il a bénéficié d'une rééducation pour des pieds bots.

**Tableau 6:** Évolution des prématurés en post-partum

Diagnostic	Terme d’accouchement	Voie d’accouchement / Indication VH	Evolution
	Prématurés	Prématurés	Prématurés
DRMK Gche +système double+suspicion T21	34 SA	VB	Evolution favorable Pas de T21
			Pieds bots (Rééducation)
			**Echo** : DRMK Gche - Pas de système
DRMK Gche + système double + urétérocèle	36 SA + 3j	VH / RPM	Hospitalisation en néonatologie : IR
			**Décès J23**
DRMK bilatérale	34 SA	VB	**Décès H10**

**VB**: Voie basse; **VH**: Voie haute; **RPM**: rupture prématurée des membranes; **IR**: insuffisance rénale; **SA**: semaine d’aménorrhée; **gche**: gauche

**Evolution des grossesses menées à terme**: (Tableau 7).

**Tableau 7:** Évolution des grossesses menées à terme

Diagnostic	Voie d’accouchement / Indication VH	Evolution néonatale
DRMK Dte + Urétéro hydronéphrose Gche	VH/oligoamnios sévère	IR + IU
		**Scintigraphie:** rein Gche hypoperfusé assurant la totalité de la fonction rénale + rein Dt muet
		**Chirurgie :** urétérostomie +Réimplantation
DRMK Dte	VB	Hospitalisation en néonat
		FR normale
		Ectopie testiculaire homolatérale à la dysplasie (opérée)
		**Scintigraphie :** bon résultat
DRMK Gche + VUP + mégavessie	VB	**Echo post natale:** DRMK Gche + Rein Dt discrètement hyperéchogène siège de kystes corticaux millimétriques.
DRMK bilatérale + Système dble + VUP	VH / SFA	Paraphimosis
		**Perdu de vue** en postnatal
DRMK Gche + agénésie rénale Dte	VB	**Décès**
		**Echo post mortem:** rein Gche en position anormale et dysplasique, agénésie rénale Dte confirmée, bassinet dilaté
DRMK bilatérale + Sd Prune Belly + VUP	VH / SFA	**Détresse respiratoire**
		**Echo post natale:** DRMK bilatérale, vessie à paroi épaisse, VUP
		**Décès à H6**
DMK Ghe +Système dble+ VUP+ Hydrocèle bilat	VB	Perdu de vue
DRMK bilatérale + Polymalformation	VB	**Décès H4**
		**Fœtopathologie** non concluante
DRMK bilatérale + VUP	VH / Anamnios	**ECBU** demandé
		**Echo post natale:** urétéro hydronéphrose bilatérale laminant le parenchyme rénal par endroit avec un contenu échogène. Qq images kystiques corticale rénale. Vessie hypertrophiée diverticulaire semi pleine a contenu échogène

**SFA:** souffrance fœtale aigue; **VUP:** valve de l’urètre postérieur; **VB:** voie basse ; **VH:** Voie haute; **RPM:** rupture prématurée des membranes; IR:insuffisance rénale; **IU:** infection urinaire; FR:fonction rénale

## Discussion

La dysplasie rénale multikystique (DRMK) est une des plus fréquentes malformations de l'appareil urinaire, regroupées sous le terme de “Congenital Abnormalities of Kidney and Urinary Tract (CAKUT)”, ou anomalies congénitales des reins et des voies urinaires. Environ 40% des insuffisances rénales terminales de l'enfant sont secondaires aux CAKUT [1]. Certaines formes de DRMK peuvent être familiales, mais la plupart sont sporadiques [2, 3]. Elle est néanmoins exceptionnellement responsable d'insuffisance rénale terminale, le pronostic dépendant du rein controlatéral. Actuellement, la quasi-totalité des DRMK soit 94,1% sont diagnostiquées par l'échographie prénatale, généralement lors de l'examen morphologique réalisé entre 20 et 22 semaines d'aménorrhée SA [4]. Dans ce contexte, l'obstétricien et l'échographiste sont souvent confrontés à de nombreuses interrogations de la part des parents, notamment en termes de pronostic et de prise en charge à court et long terme [5]. Dans la vision classique, la DMK résulterait d'un défaut d'induction du blastème métanéphrique par le bourgeon urétéral. Une autre hypothèse suggère que la DRMK serait secondaire à une anomalie majeure de l'écoulement des urines fœtales survenant précocement dans le développement rénal. Par conséquent, toute urine fœtale secrétée dans un système excréteur obstrué entraînerait un risque dysplasique d’autant plus important que l’obstruction est précoce comme les formes sévères de valves de l’urètre postérieur et d’urétérocèles obstructives sur duplicité. Ces deux théories éclairent bien des constatations cliniques mais ne peuvent tout expliquer et de nombreuses autres théories leur ont été opposées. A l’évidence, la DRMK est multifactorielle [5]. Par ailleurs, des facteurs génétiques ont également été incriminés dans la genèse de la DRMK comme certains gènes: PAX2, SIX2, BMP4, TCF2. L'association d'une DMK unilatérale à une anomalie controlatérale du parenchyme rénal, en particulier d'une hyperéchogénicité rénale ou de microkystes, doit faire rechercher une mutation de TCF2. La DRMK peut également s'inscrire dans le cadre d'une anomalie chromosomique (trisomie 21, syndrome de Turner…) ou syndromique (syndromes de Di George, de Kallmann, de Meckel Grüber…). Les affections kystiques du rein sont rares et ne représentent que 1% des affections urologiques de l'enfant, néanmoins la DRMK reste la plus fréquente des affections de l'appareil urinaire. Selon une étude faite au sein du Service de Gynécologie et Obstétrique II du CHU Hassan II de Fès, cette pathologie représente 44,44 % des uronéphropathies malformatives (16 cas de DRMK rapportés sur les 36 étudiés) [6]. Alors que dans l'étude de Fu *et al.* [7] dysplasie rénale multikystique représente 19,5% des malformations de l'appareil urinaire entrant dans le cadre des CAKUT. La DRMK atteint le plus souvent les garçons dans 60% des cas, avec un sexe ratio de 1,48 [8] ce que confirment toutes les études. Dans notre série, on a noté une très large prédominance du sexe masculin avec un pourcentage de 71%. On a évalué le sexe ratio à 2,6/1 qui dépasse largement la moyenne rapportée qui peut être expliqué par le nombre restreint de cas que comprend notre étude comparé aux autres centres de référence.

Alors que certaines études suggèrent une routine du troisième trimestre après la 32^ème^ semaine de gestation pour un meilleur taux de détection de la DRMK [9] beaucoup recommandent la fenêtre du deuxième trimestre pour ne pas manquer les cas qui montrent une involution prénatale [10]. Dans la DRMK, les kystes rénaux sont de taille inégale, juxtaposés, non communicants, à paroi fine. Les kystes sont répartis de façon aléatoire, donnant un aspect irrégulier aux contours du rein, lui faisant perdre sa forme normale. Le pyélon n'est pas visible. Le tissu entre les kystes est habituellement hyperéchogène et cette hyperéchogénicité est souvent le premier signe visible de dysplasie. La différentiation cortico-médullaire est absente. L'artère rénale en Doppler est grêle voire non présente. Tous ces arguments donnent à l'échographie une sensibilité proche de 100% pour le diagnostic de DRMK. En cas de dysplasie sur obstruction haute (Sd de jonction pyélo-urétérale), on observera souvent des gros reins contenant de volumineux kystes, alors qu'en cas de dysplasie sur valve de l'urètre postérieur, on observera plutôt des petits reins échogènes contenant de petits kystes peu nombreux [11]. Si l'atteinte est unilatérale, le rein controlatéral peut s'hypertrophier de façon compensatrice et une petite hydronéphrose est habituellement visible sans que ceci soit péjoratif. Mais, dans près de 40% des cas, on trouvera une pathologie controlatérale. En dépit du fait que le volume rénal est le paramètre de mesure le plus précis [12], il est largement accepté d’utiliser la longueur rénale à la place parce que c’est le diamètre le plus reproductible. Un des problèmes de mesure de la taille rénale est le fait qu’il est parfois difficile de distinguer les reins et les glandes surrénales. La mesure prénatale de la taille du rein sain controlatéral, dans l’espoir de l’utiliser comme référence, s’est révélée inefficace vue l’hypertrophie compensatrice qu'occasionne la dysplasie [13]. La DRMK est une anomalie le plus souvent unilatérale du développement rénal, tandis que l'atteinte bilatérale est rare, alors généralement létale [14] (102). Le côté gauche est atteint dans 53 % des cas (8). Dans l'étude d' Al Naimi *et al.* [15], l'atteinte unilatérale prédominait avec 61% et logeait plus souvent au niveau du rein droit contrairement à ce que rapporte la littérature. Comme rapportaient les auteurs Pei-Yang Hsu *et al.* avec 87% pour la DRMK unilatérale et la prédominance du côté droit avec un taux de 56%. Par contre, dans notre série, la DRMK touchait plus souvent les deux reins avec un taux de 61%. Mais quand elle était unilatérale, elle se localisait dans 71% des cas au niveau du rein gauche comme le rapporte la littérature.

Bien qu'il s'agisse le plus souvent d'une anomalie unilatérale, la découverte d'une DMK doit faire rechercher soigneusement une anomalie associée du rein controlatéral ou des organes génitaux internes. Le rein controlatéral peut présenter des anomalies diverses telles un reflux vésico-urétéral (RVU), un syndrome de la jonction pyélo-urétérale ou une dysplasie. Par ailleurs, certaines études ont rapporté des malformations extrarénales associées à la dysplasie rénale multikystique comme l'atrésie oesophagienne ou duodénale, le méningocèle ou encore des cardiopathies. La prévalence élevée des anomalies urogénitales chez les patients porteurs de DRMK peut être expliquée par l'interdépendance embryonnaire entre l'ébauche du rein, des uretères et de l'appareil génital. Jusqu'à 39% des patients présentent des anomalies associées du rein controlatéral. Une étude récente portant sur 97 enfants avec DMK retrouve 20% d'anomalies controlatérales (dilatation pyélocalicielle principalement) [16]. Dans notre série, 62,5% des DRMK unilatérales présentaient une atteinte du rein controlatéral et 72,22% avaient une anomalie associée. La dilatation urétéropyélocalicielle est l'anomalie la plus rapportée avec un pourcentage de 39%. Pour ce qui est des anomalies extra-rénales, notre étude en a retrouvé chez 28% des fœtus. Les malformations au niveau du cerveau étaient les plus fréquentes avec 17% suivies des atteintes cardiaques (11%) et de la face et du cou (11% également). La quantité de liquide amniotique doit être aussi étudiée. Elle doit être de volume normal en cas de DRMK unilatérale sauf en cas de lésions du rein controlatéral responsable alors d'oligoamnios [17]. Le pronostic de l'oligoamnios se joue à deux niveaux: 1) au cours du développement fœtal tout d'abord: en effet, l'oligoamnios expose à un risque accru d'hypoplasie pulmonaire consécutive à la compression de la cage thoracique ainsi qu'à un déficit de liquide intra-trachéal [18]; 2) à l'accouchement ensuite: avec l'accroissement du nombre d'interventions (déclenchements, césariennes) en rapport avec une augmentation de la fréquence des RCIU, des anomalies du rythme cardiaque fœtal (RCF) et des malformations qui peuvent y être associés. Quand le diagnostic est posé, une surveillance échographique anténatale est nécessaire afin de: 1) juger le profil évolutif de la DMK qui peut être stable, régresser ou progresser au cours de la vie fœtale; 2) évaluer la fonction et la morphologie du rein controlatéral, si c'est unilatéral, pour pouvoir établir un pronostic; 3) surveiller l'apparition de nouvelles anomalies rénales ou extra-rénales; 4) quantifier le liquide amniotique; 5) quoi que le rythme de surveillance ne soit pas bien codifié dans la littérature, la surveillance dans notre service est basée sur des échographies obstétricales répétées d’une manière mensuelle à bimensuelle pour rechercher des signes d'aggravation de la fonction rénale. D'après les études, la plupart des DRMK ont tendance à involuer et à diminuer de taille en anténatal, que ce soit complètement ou partiellement [19].

L'involutiontotale a été montrée dans 20% et l'involution partielle dans 67% des DRMK [20]. Dans notre série, les parturientes avaient bénéficié d'un contrôle échographique régulier où on a pu surveiller la taille et le nombre des kystes, le rein controlatéral ainsi que le liquide amniotique. L'évolution a été marquée par la stabilisation des atteintes dans 44,44% et l'aggravation de la dysplasie rénale dans 38,9% soit avec l'augmentation de la taille et du nombre des kystes (16,7%), l'apparition de kystes au niveau du rein controlatéral (22,22%) ou encore la diminution de la quantité du liquide amniotique (16,7%). Quatre cas de DRMK unilatérale et 3 cas de DRMK bilatérale ont connu une évolution défavorable. La dysplasie rénale multikystique est réputée d'avoir un bon pronostic lorsqu'elle est unilatérale et isolée. Cependant, plusieurs paramètres sont susceptibles d'influencer ce pronostic à savoir: a) l'association d'autres anomalies rénales; b) l'atteinte du rein controlatéral; c) l'augmentation du nombre de kystes; d) apparition de kystes au niveau du rein controlatéral; e) la présence de malformations extra-rénales associées; f) l'atteinte syndromique ou polymalformatif; g) l'anamnios. Alors que la littérature rapporte un pronostic péjoratif pour la forme bilatérale la décrivant comme létale, notre étude a recensé deux cas de DRMK bilatérale qui ont survécu en post partum: a) le premier enfant est porteur d'une dysplasie multikystique au niveau du rein droit et du pôle supérieur du rein gauche. Agé à ce jour de 5 ans, il ne présente aucune complication. b) Le deuxième est un nouveau-né qui a été hospitalisé en néonatologie pour insuffisance rénale et dont la fonction rénale s'est améliorée progressivement âgé à ce jour de 5 mois. L'étude de Lopez [21] sur un cas de grossesse gémellaire, a suivi de près le cas d'un des jumeaux qui présentait des lésions rénales bilatérales: un rein droit multikystique et une dysplasie multikystique segmentaire du pôle supérieur du rein gauche sur duplicité complète. L'évolution a été marquée par la normalisation de la fonction rénale et la régression du nombre des kystes (3 kystes à l'âge de 1 an). Donc, une atteinte bilatérale des reins peut être compatible avec un développement et une fonction rénale normale lorsque la DRMK n'intéresse que l'un des pôles rénaux.

## Conclusion

La dysplasie rénale multikystique est une vaste catégorie de malformations congénitales pouvant être secondaire à une uropathie obstructive. Son diagnostic anténatal repose essentiellement sur l'échographie obstétricale. Grâce aux progrès qu'elle a connu et à la qualification des échographistes, le dépistage peut se faire précocement. La recherche des anomalies associées, rénales ou extra-rénales, est indispensable vue la possibilité d'anomalie chromosomique ou génique. Le pronostic de la DRMK bilatérale reste imprévisible à cause de l’incapacité à quantifier le parenchyme fonctionnel résiduel alors que celui de la DRMK unilatérale demeure bon lorsqu'elle est isolée. Cette étude relate l'expérience du service de Gynécologie-Obstétrique 2 au CHU Hassan II de Fès en mettant en évidence toutes les avancées en matière de dépistage, de surveillance échographique et de prise en charge périnatale des dysplasies rénales multikystiques par ailleurs l'effectif de notre série est faible par rapport à celui des études avec lesquelles nous avons comparé nos résultats. De plus, aucun cas n'a bénéficié d'étude biologique, génétique ou radiologique (IRM) en anténatal par faute de moyens et de couverture sociale. Nous ne disposons pas également d'un recul suffisant pour établir un pronostic adéquat et garantir l'évolution de ces patients, ce qui met en évidence certaines failles qui persistent dans notre système de santé.

### État des connaissances actuelles sur le sujet

- La dysplasie rénale multikystique (DRMK) est une des plus fréquentes malformations de l'appareil urinaire, regroupées sous le terme de “Congenital Abnormalities of Kidney and Urinary Tract (CAKUT)”;
- Le diagnostic anténatal repose sur l'échographie obstétricale;
- Le but du diagnostic anténatal et de poser le diagnostic, rechercher les malformations associées et d'établir un pronostic.

### Contribution de notre étude à la connaissance

- Effectif de notre série est considérable pour un pays africain en l'absence de centre de référence en diagnostic anténatal;
- La possibilité de mettre sur pieds le diagnostic des DRMK tout en essayant de prédire le pronostic et de prévoir une prise en charge post-natal approprié dans un contexte de ressources limitées.

## Conflits d’intérêts

Les auteurs ne déclarent aucun conflit d'intérêts.
